# Autologous Fat Transfer Procedure Complications in Four Emergency Departments in Miami, Florida

**DOI:** 10.5811/westjem.54037

**Published:** 2026-07-16

**Authors:** Mary Bridget Lee, Joshua Goldstein, Daniel Hercz, Matthew Pyle

**Affiliations:** * Montefiore Medical Center, Department of Critical Care Medicine, Bronx, New York; † Massachusetts General/Brigham Hospital System, Department of Pulmonary & Critical Care Medicine, Boston, Massachusetts; ‡ PeaceHealth SW, Department of Emergency Medicine, Vancouver, Washington; § Jackson Health System, Department of Emergency Medicine, Miami, Florida; || University of Miami, Miller School of Medicine, Department of Surgery, Miami, Florida

## Abstract

**Introduction:**

Our study objective was to share the experiences of four public emergency departments (ED) caring for patients with complications of gluteal autologous fat transfer (AFT), colloquially known as Brazilian butt lifts, to provide emergency physicians with a better understanding of these patients and potential complications associated with the procedure.

**Methods:**

Jackson Health System in Miami, Florida, undertook an institutional initiative to prospectively collect data on patients presenting to any of the system’s EDs with gluteal AFT complications from October 2020–May 2023. We abstracted patient charts for demographics, disposition, lab values, diagnoses, and care-related charges to generate descriptive statistics. Our primary outcome measure was diagnostic code and diagnostic category. Secondary outcome measures were hemoglobin on presentation, units of blood transfused, admission level of care, hospital charge, length of stay, and mortality.

**Results:**

Of 234 potentially relevant patient charts identified by our hospital protocol, 157 patients met our criteria. All were female, with an average age of 33. A total of 123 patients were from out of state, and one was international. The most common diagnoses were anemia/bleeding (81 patients), followed by pain (65), cardiac (36) and hypovolemia/dehydration (32). Our analysis revealed that 86 patients presenting to the ED required admission (54.8%), with 12 patients requiring intensive care unit care (7.6%). There was one death (0.6%). Total hospital charges for this cohort equaled $2,822,540, averaging $17,977 per patient.

**Conclusion:**

Gluteal AFT procedures are associated with a wide range of complications, morbidity, and mortality. These patients have a high admission rate and the potential to be critically ill. Patients travel from around the country to have these procedures performed, and it is crucial that EDs nationally are prepared to care for them.

## INTRODUCTION

### Background

Gluteal autologous fat transfer (AFT) procedures, colloquially known as “Brazilian butt lifts,” are gaining prominence in the aesthetic surgery community. A surgical technique to augment a patient’s buttocks, AFT involves abdominal liposuction followed by re-injection of the fat into the gluteal area. The procedure has soared in popularity as beauty standards have shifted and cosmetic surgery has become increasingly transparent on social media platforms, with reported cases in the United States increasing from 614 in 2002 to 61,387 by 2021, a nearly 10,000% increase. 1–3 The procedure can cost thousands of dollars and requires significant postoperative care; in addition to typical postsurgical needs, patients often require aggressive lymphatic massage and have significant movement restrictions that include sleeping face down, avoiding sitting, and wearing compression garments. 4

### Importance

Plastic surgeons have been investigating complications of gluteal AFT for more than a decade. 5–8 The most dangerous complication of gluteal AFT is pulmonary fat embolism. This type of embolism may also occur when fat is injected or absorbed into a blood vessel during the re-injection phase of an AFT procedure. It has even been suggested that moving the patient into a supine position postoperatively can put enough pressure on newly deposited adipose tissue to shift it into the bloodstream. 7 Theoretically, ultrasound guidance should decrease this risk, which formed the basis for a 2022 Florida Board of Medicine regulation requiring its use. 9 Pulmonary fat emboli from gluteal AFT are rare (1:1,030 procedures) but life-threatening. Mortality rates are as high as 50% 6,10 —the highest for any aesthetic procedure. 6

Miami, Florida, has become a popular destination for these procedures. Unfortunately, as cases have increased, South Florida has also seen an increase in well-publicized poor outcomes. When seven gluteal AFT patients died in one year alone, the Florida Board issued an emergency regulation restricting fat injection to the subcutaneous space as opposed to injecting into muscle. 11 Despite this new regulation, at least 13 deaths were reported in Miami-Dade County from 2019–2022, the three years immediately following the implementation of the subcutaneous injection regulation. 7 These deaths led to another wave of regulations, the impact of which has yet to be reported.

### Goals of the Investigation

Because a significant number of gluteal AFT procedures are performed at standalone outpatient surgical centers, accurate data on complication rates are difficult to obtain. The existing literature, which is aimed primarily at specialists in plastic and cosmetic surgery, relies on voluntary reporting, physician surveys, and postmortem reports.

Jackson Health System (JHS) is a nonprofit academic health system operated by Miami-Dade County and affiliated with the University of Miami Medical School. The system operates four main hospitals, one pediatric hospital, and a Level I trauma center. Three smaller JHS emergency departments (ED) are located in suburban Miami-Dade County: Jackson South (41,000 visits per year); Jackson West (27,000;) and Jackson North (53,000). Jackson Memorial, the larger, tertiary-care facility in downtown Miami has 112,000 visits per year. Many cases are transferred from other facilities to Jackson Memorial for subspecialist evaluation or when a higher level of care is required. These hospitals are well positioned to add quantitative data regarding emergency care of gluteal AFT procedures to the existing literature.

**Population Health Research Capsule**

What do we already know about this issue?

*Gluteal autologous fat transfer (AFT) procedures are associated with high mortality rates compared with other aesthetic procedures*.

What was the research question?

*What are the patient characteristics and medical complications of gluteal AFT procedures presenting to the emergency department (ED)?*

What was the major finding of the study?

*The top diagnostic categories were anemia (51.6%), pain (41.4%), cardiac (22.9%) and hypovolemia (20.4%)*.

How does this improve population health?

*This study provides the first description of this patient population in an ED setting and provides emergency physician-specific guidance for their care*.

In this paper we aimed to describe the experience of caring for patients with gluteal AFT complications in the ED setting. Our goal was to fill a gap in the medical literature to assist emergency physicians nationwide in treating these patients who have had surgeries performed in their own cities or returned home following “medical tourism” trips.

## METHODS

### Study Design and Setting

We reviewed the 12 criteria for emergency medicine medical record review studies, as per Worster et al, and addressed them as described below.^12^ This was an institutional review board-approved cross-sectional chart review of patients presenting to JHS EDs with complications of gluteal AFT procedures during the 32 months between October 2020–May 2023 (Worster criteria 12). The Public Health Trust of Miami-Dade County operates four hospitals in the JHS that serve as the medical safety net and major referral center for the region. Our health system also partners with the University of Miami School of Medicine in its capacity as a teaching hospital. The hospital system uses the Cerner electronic health record system (Oracle Health, Nashville, TN).

### Selection of Participants (Worster Criteria 2 and 10)

In 2020, JHS undertook an institutional initiative to identify patients with gluteal AFT procedure complications. Nursing leadership created a patient safety event that enabled anonymous reporting by anyone involved in the patient’s care. An electronic form accessed through our in-house patient safety reporting system prompted clinicians to provide the patient’s medical record number and date of visit, as well as optional fields that included patient demographics, the nature of the complication, and the name of the facility where the procedure had been performed. Reminders were sent via institution-wide emails and announced at meetings where clinical staff were encouraged to submit the information whenever they had a patient who met the inclusion criteria: patients presenting to the ED after a gluteal AFT with a complication presumed to be secondary to the procedure.

Study investigators JG and MBL, working independently, manually reviewed all Cerner charts identified during the study period for exclusion criteria. Charts were excluded if the patient was < 18 years of age at the time of the visit, pregnant, incarcerated, or required a guardian or healthcare proxy due to cognitive impairment. We also excluded charts if the visit was not preceded by a gluteal AFT procedure (defined as liposuction followed by fat transfer to the gluteal area).

### Measurements

Investigators reviewed Cerner medical record charts that met inclusion criteria and collected data regarding patient demographics, diagnostic codes, vital signs, medical interventions, blood transfusions, disposition, level of care, hospital length of stay, and mortality (Worster criteria 3 and 9). Missing demographic information was reported as unknown. Five patients did not have lab results; these patients were excluded from analysis related to hemoglobin level. No other missing data was encountered (Worster criteria 11). All charts included up to five *International Classification of Diseases*, 10^th^ ed (*ICD-10*) diagnostic codes (primary through quinary) assigned to the encounter. JG and MBL performed the data collection and abstraction with the use of a standardized, closed-response spreadsheet with pre-defined variables (Worster criteria 4). Study investigator MP adjudicated any discrepancies between the two primary abstractors (Worster criteria 5). Each patient was assigned a study number, and all identifying information, including name, birthdate, and medical record number, was removed from the data.

Abstractors were not blinded to the study hypothesis and did not receive formal training prior to data collection (Worster criteria 1 and 6). Interobserver reliability testing was not possible in the selection of the initial dataset as the medical record numbers were provided to us by the Office of Patient Safety based on the data from the event reporting program. However, two authors subsequently reviewed each chart individually to assess whether the patient met the predefined inclusion criteria and then extracted the relevant clinical data. There was 100% agreement on the 77 cases that did not meet inclusion criteria and were excluded; and intraclass correlation coefficient was > 0.75 for all clinical data points extracted (Worster criteria 7 and 8).

We performed descriptive statistics on this de-identified data to reach our conclusions regarding the presentation and care of these patients. Often, similar (but not identical) diagnostic codes were used across the study population. Due to this variation in code assignment, reporting the prevalence of specific diagnostic codes would not have accurately reflected the prevalence of symptoms and disease states. Therefore, related diagnostic codes were grouped into diagnostic categories. All *ICD-10* codes were reviewed and categorized by relevant body system. The *ICD-10* diagnoses with the same or similar keywords were grouped together (eg, all *ICD-10* codes with “anemia,” “hemorrhage,” or “blood loss” were categorized under “anemia”). The *ICD-10* codes with “history of” language were not included in the categorization. Codes that were not related to a specific body system, or lacked similar keywords, were not placed in a category.

Our billing department provided cost analysis after patient discharge. For the purposes of this analysis we calculated only charges, rather than actual payments.

## RESULTS

**Figure 1:**
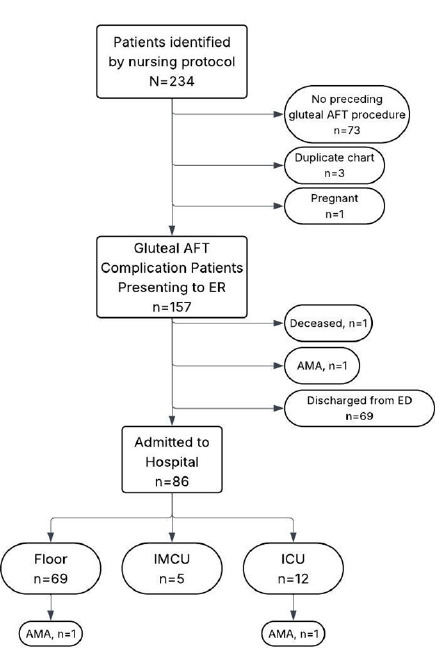
Study flow diagram of patients presenting to Miami-area emergency departments with complications following gluteal autologous fat transfer procedures. *AFT*, autologous fat transfer; *AMA*, against medical advice; *ED*, emergency department; *IMCU*, intermediate care unit; *ICU*, intensive care unit.

Nursing leadership provided investigators the 234 charts that had been flagged by the referral protocol during the study period. Three patients had more than one ED visit for complications related to the same procedure. These multiple encounters were treated as one entry. One patient had a positive pregnancy test at the time of her ED visit. We excluded 73 charts that did not reference a gluteal AFT procedure. In total, 77 charts were excluded, leaving 157 charts for analysis. See Figure 1.

### Characteristics of Study Subjects

**Figure 2:**
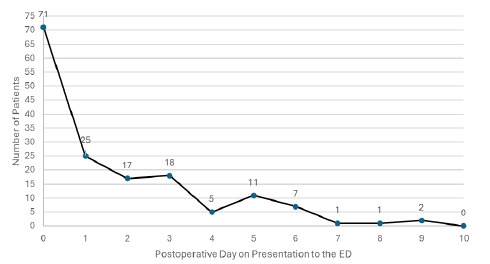
Time to emergency department presentation from a retrospective chart review of patients presenting to Miami-area EDs with complications related to gluteal autologous fat transfer procedures. *ED*, emergency department.

The average patient age was 33 (standard deviation 9.5). All patients (100%) were women, including one transgender woman. Patient charts identified 83 White women (52.9%), 71 Black women (45.2%), and three women (1.9%) whose race was listed as “Other.” In the study cohort, 25 (15.9%) were described as Hispanic or Latina in the medical record. There were 123 patients (78.3%) with a primary address out of state; one patient had an international address (0.6%); and four had no home address recorded (2.6%). Patients presented on median postoperative day 1 (IQR 3). The cohort included one patient presenting in 2020 (0.6%), 39 in 2021 (24.8%), 85 in 2022 (54.1%), and 32 in 2023 (20.4%). See Table 1 and Figure 2.

### Main Results

#### Disposition

The ED admitted 86 gluteal AFT patients (54.8%). Of those admitted, 69 (80.2%) were admitted to the medical floor, five (5.8%) to the intermediate care unit (IMCU), and 12 (14.0%) to the intensive care unit (ICU) (Figure 1). Average length of stay was 1.8 days (SD 2.2). Three patients left against medical advice.

#### Medical Complications

Patient visits often had more than one diagnostic code assigned. The most common diagnostic codes were related to anemia and bleeding (81 charts [51.6%]). The average initial hemoglobin level was 7.9 grams per deciliter (SD 1.9). Within the first 24 hours of presentation, 54 patients (33.1%) required a blood transfusion. Of those transfused in the first 24 hours, an average of 1.6 units (0.9) were transfused. A total of 63 patients (40.1%) required a blood transfusion at some point during their hospitalization.

There were 65 patient charts (41.4%) with *ICD-10* codes associated with pain, 36 (22.9%) with cardiac-related *ICD-10* codes, and 32 (20.4%) with diagnostic codes related to hypovolemia. Five patients required an operative intervention during their hospitalization for two hematoma evacuations, two bowel perforations, and one incarcerated ventral hernia. See Table 2 for additional information.

#### Severe Complications

After their procedures several patients suffered severe complications, defined as requiring an ICU admission or a cardiac arrest *ICD-10* code. The reasons for ICU admission are listed in Table 3. The average age of patients with severe complications was 40.5 years (SD 8.1). Patients presented a median of one day post-procedure (IQR 2). Of the 12 patients admitted to the ICU, three required an operative intervention. The average presenting hemoglobin among the ICU patients was 8.8 (2.3). Seven ICU patients required a blood transfusion in the first 24 hours and nine at any point during their hospitalization. The nine patients who required a transfusion received an average of 3.2 units (1.7). The most common diagnostic categories for patients with severe complications were anemia (50%), shock (41.7%), respiratory (41.7%), cardiac (41.7%), and renal (33.3%). See Appendix A for details regarding diagnostic categories.

Three patients had a cardiac arrest *ICD-10* code. Of these, two had a cardiac arrest prehospital and one had an intra-operative cardiac arrest after admission. One of the prehospital cardiac arrest patients died. There were no other deaths in the study cohort.

#### Cost

The total hospital charges for these patients amounted to$2,822,540. Gluteal AFT complication care charges averaged $17,977 per patient (IQR $14,224).

## DISCUSSION

South Florida has been described as a hub for gluteal AFT procedures both in popular media and in the medical literature.^2^,^4^,^7^ Our study identified 157 patients who presented to JHS EDs with gluteal AFT procedure complications in a 32-month period. Because the study was limited to a single hospital system we could not determine whether the size of this cohort was disproportionate to other geographic locations. However, most patients appeared to have traveled from out of state for their procedures, suggesting that Miami is a destination for gluteal AFT procedures. Although patients typically presented within three days of their procedures, we saw patients presenting with gluteal AFT complications up to postoperative day 9. This suggests that some patients likely returned to their home states within the complication period and reinforces the importance of emergency clinician awareness of the procedure and its potential complications, regardless of their geographic location.

The most common presenting complaints were related to bleeding, pain, and cardiac abnormalities. Patients were anemic on presentation with an average hemoglobin of 7.9. One-third of patients required a blood transfusion within 24 hours of presentation. Patients admitted to the ICU had higher presenting hemoglobins but were more likely to require a blood transfusion and more units of blood, perhaps explained by the higher rate of return to the operating room in this group.

Postoperative pain emerged as another common concern. This was distressingly illustrated by a patient who overdosed on her home methadone in an attempt at better pain control. Safely treating postoperative pain improves patient experience and may reduce ED visits. The cardiac abnormalities that patients experienced represented a broad spectrum from sinus tachycardia to cardiac arrest. It is likely that some of the less-severe cardiac diagnoses were secondary to bleeding, pain, and other primary concerns.

A recent study by Weidman et al looked at complications of outpatient fat grafting and gluteal augmentation identified in QUAD A (American Association for Accreditation of Ambulatory Surgery Facilities). Although these complications were self-reported to a regulatory body after procedures performed in accredited facilities, the authors did find that complication rates doubled from 2020 to 2021, which represents the beginning of our study period. Of the 436 complications reported, wound infection was the most common (22.3%).^13^ Similarly, a 2023 systematic review of 1,788 gluteal fat augmentation procedures found seroma (6.9%) and infection (3.0%) to be the most common complications.^14^ Interestingly, we had much lower rates of infectious complications in our patient population. Given that the patients in our study presented on median postoperative day 1, it is possible our reporting protocol overidentified patients presenting early in their postoperative course.

Another study looked at complication rates of different autologous gluteal augmentation techniques in 185 patients and reported no major complications.^15^ The patients in that study underwent the procedure to correct truncal deformity after significant weight loss; the procedure itself differs from the gluteal AFT in several ways, notably the creation of a cutaneous flap. The absence of major complications in that study’s patient group may suggest that the patients in our review underwent a more invasive procedure, with environmental or technical differences that put them at greater risk.

Mortality associated with gluteal AFT procedures has increasingly come under scrutiny in the past few years. A 2017 report by the Aesthetic Surgery Education and Research Foundation (ASERF) estimated the incidence of mortality to be between 1:6,214 to 1:2,351; however, ASERF noted that the estimates were significantly limited by a lack of standardized reporting of complications.^6^ In 2019, ASERF conducted a survey of plastic surgeons, with responses suggesting a decrease in the occurrence of pulmonary fat emboli and incidence of mortality to 1:2,493 and 1:14,952, respectively.^16^ This would indicate that gluteal AFT is now safer than abdominoplasty (incidence of mortality, 1:13,147).^6^ However, absolute number of deaths from pulmonary fat emboli following gluteal AFT increased in South Florida after this report.^7^ Much of the existing literature on gluteal AFT complications focuses on pulmonary fat embolism; no studies have reported the number of patients presenting to the ED after a gluteal AFT procedure. The results of our study indicate that most patients presenting to the AD with gluteal AFT complications met admission criteria but were hemodynamically stable, with cases of critical illness relatively uncommon.

Pulmonary fat embolism is the most widely discussed serious complication of gluteal AFT in the media and scientific literature; however, this diagnosis can only be made at postmortem examination. Two patients in our cohort had presentations highly concerning for pulmonary fat embolism but did not receive a definitive diagnosis. Both patients decompensated during or immediately after their gluteal AFT procedures: one had a cardiac arrest and was declared dead on ED arrival, and one developed stroke-like symptoms. At the time of publication, an autopsy report for the deceased patient was not available. It is difficult to compare mortality and presumed pulmonary fat embolism for our cohort to that reported in the literature given the different denominators. The incidence of mortality reported by ASERF is based on estimates of the total number of gluteal AFT procedures performed, whereas our study is based on number of patients presenting to the ED. For two patients, the timing of their critical illness appears to have been coincidental; one patient developed new-onset diabetes with diabetic ketoacidosis, and one patient developed a perforated marginal ulcer at the site of a prior bariatric surgery. For these patients there was no clear mechanism of injury related to the gluteal AFT procedure, although a connection cannot be ruled out.

Patients with gluteal AFT complications accumulated significant hospital charges related to their care. The hospital charges equaled $2,822,540, or an average of $17,977 per patient. To our knowledge, we are the first to report on the financial implications of gluteal AFT complications. Reducing complication rates and appropriately evaluating these patients in the ED could reduce the financial burden on both patients and health systems.

We offer several recommendations to emergency physicians based on these findings. First, and most broadly, even though many of these patients are young and may have been in good health prior to their surgery, they have the potential to be critically ill; therefore, a high index of suspicion should be maintained in their evaluation. These patients are not suitable for “fast track” or low-acuity areas of the ED. Second, given the significant overlap in how the most common categories of complaints (pain, hypovolemia, and anemia) can present both clinically and hemodynamically, clinicians must take care not to anchor on any one cause and to recognize (and manage) the possibility of multiple simultaneous processes.

Third, given the high number of patients in our study who required blood transfusion, particular attention should be paid to clinical signs and symptoms of hemorrhagic shock or bleeding. Patients should have type and crossmatching sent as part of their initial lab evaluation, and clinicians should also be aware of the potential need for cross-sectional imaging, specifically angiography, to locate the source of the bleeding. Finally, clinicians should be aware of the specific challenges with these patients: they are frequently reluctant to remove their postoperative garment; they have often had their postoperative pain undermanaged; and they frequently travel for this procedure, which heightens their emotional and psychological distress because they were far from home while acutely ill—all of which can delay or complicate the initial evaluation.

## LIMITATIONS

Our greatest methodological challenge was identifying all patients presenting with gluteal AFT complication from the medical record. There is no *ICD-10* code specific for this diagnosis or for cosmetic surgery complications generally. These patients are assigned an array of different codes based on physician judgment, making a retrospective chart search difficult. Rather than manually reviewing all charts with general surgical complication codes, we were able to use internal data collected under a new hospital protocol. Accurate data collection depended on the care team participating in the reporting protocol (ie, our findings may not represent a comprehensive list of all gluteal AFT complications that presented during the study period). The time distribution of patients presenting to the ED was likely influenced by when staff received training and reminders about this initiative.

Additionally, because there is no standard reporting system or database for gluteal AFT procedures in Miami-Dade County, it is impossible to know with precision how many of these procedures were performed in the county during our study period. Without a denominator, we were unable to calculate the occurrence of complication for gluteal AFT procedures. For similar reasons, we could not make comparison between gluteal AFT complications and complications related to other cosmetic surgery procedures. This limited our ability to compare our findings to previously published data on this topic.

We relied on information collected during routine patient registration. As a result, some of the patient demographic information is limited. For example, our electronic health record offers three racial categories (Black, White, other), and there is no defined protocol for how these categories are determined (self-identified versus assigned). Although this results in a less-precise picture, it is sufficient to provide a basic understanding of the diversity of our patient population. Additionally, we created diagnostic categories to capture the true onset of certain symptoms and disease states. However, this is not a widely accepted and standardized process and may have introduced bias into our results.

Our goal was to increase awareness and assist emergency clinicians in caring for patients with complications of gluteal AFT procedures. However, many aspects of this procedure deserve additional research. Larger epidemiologic studies that can capture the total number of procedures performed will help elucidate the risk of complications and how it compares to other cosmetic procedures. Analysis of the impact of recent legislation regulating the performance of gluteal AFT will also help us determine what has been effective in making these procedures safer for patients. Our goal is to equip emergency physicians to care for these patients while additional research identifies ways to reduce the number of gluteal AFT complications.

## CONCLUSION

We describe a racially and ethnically diverse population that often travels out of state for cosmetic procedures. Patients present to EDs primarily for bleeding, pain, and cardiac abnormalities and have a high admission rate. A smaller subset experience severe complications, including death. We recommend that emergency physicians maintain a high index of suspicion for critical illness, evaluate for post-procedural hemorrhage, and maintain sensitivity for the unique aspects of recovering from gluteal autologous fat transfer.

## Supplementary Information



## Figures and Tables

**Table 1:** Characteristics of patients presenting to the emergency department after gluteal autologous fat transfer procedures.

Study subject characteristics	
Average age, years (SD)	33 (9.51)
Sex	
Assigned female at birth, n (%)	156 (99.36%)
Assigned male at birth, n (%)	1 (0.64%)
Gender	
Women, n (%)	157 (100%)
Men, n (%)	0 (0%)
Race	
White, n (%)	83 (52.87%)
Black, n (%)	71 (45.22%)
Other, n (%)	3 (1.91%)
Ethnicity	
Hispanic/Latina	25 (15.92%)
Non-Hispanic/Latina	132 (84.07%)
Home Address	
In state (Florida)	29 (18.47%)
Out of state (United States)	123 (78.34%)
International	1 (0.64%)
None	4 (2.55%)

*ED*, emergency department; *SD*, standard deviation.

**Table 2:** Most common complications by diagnostic code and diagnostic category in a study of patients presenting to Miami-area emergency departments after undergoing gluteal autologous fat transfer procedures.

Most Common *ICD-10* Codes			
Diagnosis	*ICD-10* Code*	Total patients, n	Percentage of all patients, %
Acute posthemorrhagic anemia	D62	53	33.76
Other acute postprocedural pain	G89.18	40	24.54
Syncope and collapse	R55	25	15.34
Anemia, unspecified	D64.9	24	14.72
Tachycardia, unspecified	R00.0	21	12.88
Unspecified abdominal pain	R10.9	14	8.59
Dehydration	E86.0	12	7.36
Hypotension, unspecified	I95.9	11	6.75
Weakness	R53.1	9	5.52
Other postprocedural shock, initial encounter	T81.19XA	8	4.91

**Most Common Diagnostic Categories**			
Diagnostic Category		Total patients, n	Percentage of all patients, %

Anemia/ Bleeding		81	51.59
Blood transfusion within 24 hours		52	33.12
Blood transfusion total		63	40.13
Pain		65	41.40
Cardiac		36	22.93
Hypovolemia		32	20.38
Electrolytes		14	8.92
Skin		14	8.92
Respiratory		12	7.64
Infection		11	7.01
Renal		11	7.01
Musculoskeletal		10	6.37

* If patients had multiple codes within the same diagnostic category, these were treated as one entry.

*ICD-10*, *International Classification of Diseases*, 10^th^ Ed.

**Table 3:** Reason for admission to intensive care unit and need for operative intervention in a study of patients presenting to Miami-area emergency departments after undergoing gluteal autologous fat transfer procedures.

Patient	Reason for ICU admission	Operative Intervention?
1	Retained hematoma, anemia	Yes
2	Hemorrhagic shock	No
3	Hemorrhagic shock	No
4	Pneumoperitoneum	No
5	Altered mental status requiring intubation for airway protection	No
6	Stroke syndrome	No
7	Cardiac arrest	No
8	Marginal ulcer perforation	Yes
9	Small bowel obstruction with perforation	Yes
10	Convulsions	No
11	Pneumoperitoneum	No
12	Diabetic ketoacidosis	No

*ICU*, intensive care unit.
