# Supplementary material for: Autologous Fat Transfer Procedure Complications in Four Emergency Departments in Miami, Florida
Source: West J Emerg Med. 2026 Jul 16;27(4):861–8. doi: 10.5811/westjem.54037 (PMC13436661; doi:10.5811/westjem.54037)
Supplement: Supplementary file 1 [file wjem-27-861-s001.docx]

| **Category** | **Diagnosis** | **ICD-10 Code** | **n** | **%** |
| --- | --- | --- | --- | --- |
| **Anemia** | | | **81** | **51.59** |
|  | Acute posthemorrhagic anemia | D62 | 53 | 32.52 |
|  | Anemia, unspecified | D64.9 | 24 | 14.72 |
|  | Other specified anemias | D64.89 | 4 | 2.45 |
|  | Iron deficiency anemia secondary to blood loss (chronic) | D50.0 | 2 | 1.23 |
|  | Iron deficiency anemia, unspecified | D50.9 | 2 | 1.23 |
|  | Hemorrhage due to other internal prosthetic devices, implants and grafts, initial encounter | T85.838A | 1 | 0.61 |
| **Pain** | | | **65** | **41.40** |
|  | Other acute postprocedural pain | G89.18 | 40 | 24.54 |
|  | Unspecified abdominal pain | R10.9 | 14 | 8.59 |
|  | Other chest pain | R07.89 | 5 | 3.07 |
|  | Generalized abdominal pain | R10.84 | 4 | 2.45 |
|  | Chest pain, unspecified | R07.9 | 3 | 1.84 |
|  | Dorsalgia, unspecified | M54.9 | 3 | 1.84 |
|  | Myalgia, other site | M79.18 | 3 | 1.84 |
|  | Headache, unspecified | R51.9 | 2 | 1.23 |
|  | Pain, unspecified | R52 | 2 | 1.23 |
|  | Pain in left lower leg | M79.662 | 2 | 1.23 |
|  | Mastodynia | N64.4 | 1 | 0.61 |
|  | Left upper quadrant pain | R10.12 | 1 | 0.61 |
|  | Myalgia, unspecified site | M79.10 | 1 | 0.61 |
|  | Pain in right leg | M79.604 | 1 | 0.61 |
|  | Pain in left leg | M79.605 | 1 | 0.61 |
|  | Pain in right thigh | M79.651 | 1 | 0.61 |
|  | Pain in left thigh | M79.652 | 1 | 0.61 |
|  | Pain in right lower leg | M79.661 | 1 | 0.61 |
|  | Chest pain on breathing | R07.1 | 1 | 0.61 |
| **Cardiac** | | | **36** | **22.93** |
|  | Tachycardia, unspecified | R00.0 | 21 | 12.88 |
|  | Palpitations | R00.2 | 6 | 3.68 |
|  | Abnormal electrocardiogram [ECG] [EKG] | R94.31 | 5 | 3.07 |
|  | Cardiac arrest, cause unspecified | I46.9 | 2 | 1.23 |
|  | Myocardial infarction type 2 | I21.A1 | 1 | 0.61 |
|  | Supraventricular tachycardia | I47.1 | 1 | 0.61 |
|  | Intraoperative cardiac arrest during other surgery | I97.711 | 1 | 0.61 |
|  | Bradycardia, unspecified | R00.1 | 1 | 0.61 |
|  | Abnormal findings on diagnostic imaging of heart and coronary circulation | R93.1 | 1 | 0.61 |
| **Hypovolemia** | | | **32** | **20.38** |
|  | Dehydration | E86.0 | 12 | 7.36 |
|  | Hypotension, unspecified | I95.9 | 11 | 6.75 |
|  | Hypovolemia | E86.1 | 5 | 3.07 |
|  | Postprocedural hypotension | I95.81 | 2 | 1.23 |
|  | Orthostatic hypotension | I95.1 | 1 | 0.61 |
|  | Other hypotension | I95.89 | 1 | 0.61 |
|  | Volume depletion, unspecified | E86.9 | 1 | 0.61 |
| **Electrolytes** | | | **14** | **8.92** |
|  | Hypo-osmolality and hyponatremia | E87.1 | 7 | 4.29 |
|  | Hypokalemia | E87.6 | 5 | 3.07 |
|  | Hypomagnesemia | E83.42 | 2 | 1.23 |
|  | Hypocalcemia | E83.51 | 1 | 0.61 |
|  | Hyperosmolality and hypernatremia | E87.0 | 1 | 0.61 |
|  | Hyperkalemia | E87.5 | 1 | 0.61 |
| **Cutaneous** | | | **14** | **8.92** |
|  | Postprocedural hemorrhage and hematoma of skin and subcutaneous tissue following other procedure | L76.22 | 5 | 3.07 |
|  | Postprocedural hematoma of skin and subcutaneous tissue following other procedure | L76.32 | 3 | 1.84 |
|  | Other postprocedural complications of skin and subcutaneous tissue | L76.82 | 2 | 1.23 |
|  | Traumatic subcutaneous emphysema, initial encounter | T79.7XXA | 2 | 1.23 |
|  | Emphysema (subcutaneous) resulting from a procedure, initial encounter | T81.82XA | 2 | 1.23 |
|  | Paresthesia of skin | R20.2 | 1 | 0.61 |
|  | Disruption of external operation (surgical) wound, not elsewhere classified, initial encounter | T81.31XA | 1 | 0.61 |
| **Respiratory** | | | **12** | **7.64** |
|  | Dyspnea, unspecified | R06.00 | 3 | 1.84 |
|  | Acute respiratory failure with hypoxia | J96.01 | 2 | 1.23 |
|  | Atelectasis | J98.11 | 2 | 1.23 |
|  | Shortness of breath | R06.02 | 2 | 1.23 |
|  | Respiratory failure, unspecified, unspecified whether with hypoxia or hypercapnia | J96.90 | 1 | 0.61 |
|  | Other forms of dyspnea | R06.09 | 1 | 0.61 |
|  | Influenza due to other identified influenza virus with other respiratory manifestations | J10.1 | 1 | 0.61 |
| **Infection** | | | **11** | **7.01** |
|  | Sepsis, unspecified organism | A41.9 | 2 | 1.23 |
|  | Fever, unspecified | R50.9 | 2 | 1.23 |
|  | Infection following a procedure, organ and space surgical site, initial encounter | T81.43XA | 2 | 1.23 |
|  | Varicella without complication | B01.9 | 1 | 0.61 |
|  | Peritoneal abscess | K65.1 | 1 | 0.61 |
|  | Postprocedural fever | R50.82 | 1 | 0.61 |
|  | Infection following a procedure, unspecified, initial encounter | T81.40XA | 1 | 0.61 |
|  | COVID-19 | U07.1 | 1 | 0.61 |
|  | Influenza due to other identified influenza virus with other respiratory manifestations | J10.1 | 1 | 0.61 |
| **Renal** | | | **11** | **7.01** |
|  | Acute kidney failure, unspecified | N17.9 | 7 | 4.29 |
|  | Urinary tract infection, site not specified | N39.0 | 3 | 1.84 |
|  | Unspecified hydronephrosis | N13.30 | 1 | 0.61 |
|  | Calculus of kidney with calculus of ureter | N20.2 | 1 | 0.61 |
| **Musculoskeletal** | | | **10** | **6.37** |
|  | Dorsalgia, unspecified | M54.9 | 3 | 1.84 |
|  | Myalgia, other site | M79.18 | 3 | 1.84 |
|  | Pain in left lower leg | M79.662 | 2 | 1.23 |
|  | Myalgia, unspecified site | M79.10 | 1 | 0.61 |
|  | Pain in right leg | M79.604 | 1 | 0.61 |
|  | Pain in left leg | M79.605 | 1 | 0.61 |
|  | Pain in right thigh | M79.651 | 1 | 0.61 |
|  | Pain in left thigh | M79.652 | 1 | 0.61 |
|  | Pain in right lower leg | M79.661 | 1 | 0.61 |
|  | Contusion of left thigh, initial encounter | S70.12XA | 1 | 0.61 |
| **Nausea & Vomiting** | | | **9** | **5.73** |
|  | Nausea | R11.0 | 3 | 1.84 |
|  | Nausea with vomiting, unspecified | R11.2 | 3 | 1.84 |
|  | Vomiting, unspecified | R11.10 | 2 | 1.23 |
| **Shock** | | | **9** | **5.73** |
|  | Other postprocedural shock, initial encounter | T81.19XA | 8 | 4.91 |
|  | Other shock | R57.8 | 1 | 0.61 |
| **Weakness** | | | **9** | **5.73** |
|  | Weakness | R53.1 | 9 | 5.52 |
|  | Other fatigue | R53.83 | 1 | 0.61 |
| **Psychiatric** | | | **4** | **2.55** |
|  | Anxiety disorder, unspecified | F41.9 | 2 | 1.23 |
|  | Major depressive disorder, single episode, unspecified | F32.9 | 1 | 0.61 |
|  | Eating disorder, unspecified | F50.9 | 1 | 0.61 |
| **PE/DVT** | | | **3** | **1.91** |
|  | Single subsegmental pulmonary embolism without acute cor pulmonale | I26.93 | 1 | 0.61 |
|  | Other pulmonary embolism without acute cor pulmonale | I26.99 | 1 | 0.61 |
|  | Acute embolism and thrombosis of left femoral vein | I82.412 | 1 | 0.61 |
|  | Acute embolism and thrombosis of unspecified deep veins of left distal lower extremity | I82.4Z2 | 1 | 0.61 |
| **GI Perforation** | | | **2** | **1.27** |
|  | Acute gastrojejunal ulcer with perforation | K28.1 | 1 | 0.61 |
|  | Perforation of intestine (nontraumatic) | K63.1 | 1 | 0.61 |
|  | Accidental puncture and laceration of a digestive system organ or structure during a digestive system procedure | K91.71 | 1 | 0.61 |
